# Supplementary material for: SARS-CoV-2 Mpro responds to oxidation by forming disulfide and NOS/SONOS bonds
Source: Nat Commun. 2024 May 7;15:3827. doi: 10.1038/s41467-024-48109-3 (PMC11076503; doi:10.1038/s41467-024-48109-3)
Supplement: Supplementary file 1 — Supplementary Information [file 41467_2024_48109_MOESM1_ESM.pdf]

Supplementary Information

SARS-CoV-2 M^{pro} responds to oxidation by forming disulfide and NOS/SONOS bonds

Patrick Y. A. Reinke[1,*], Robin Schubert[2,*], Dominik Oberthür[1], Marina Galchenkova[1], Aida Rahmani Mashhour[1], Sebastian Günther[1], Anaïs Chretien[2], Adam Round[2], Brandon Charles Seychell[3], Brenna Norton-Baker[4,5], Chan Kim[2], Christina Schmidt[2], Faisal H. M. Koua[2], Alexandra Tolstikova[1], Wiebke Ewert[1], Gisel Esperanza Peña Murillo[1, 6], Grant Mills[2], Henry Kirkwood[2], Hévila Brognaro[7], Huijong Han[2], Jayanath Koliyadu[2], Joachim Schulz[2], Johan Bielecki[2], Julia Lieske[1], Julia Maracke[1], Juraj Knoska[1,6], Kristina Lorenzen[2], Lea Brings[2], Marcin Sikorski[2], Marco Kloos[2], Mohammad Vakili[1,2], Patrik Vagovic[1,2], Philipp Middendorf[1], Raphael de Wijn[2], Richard Bean[2], Romain Letrun[2], Seonghyun Han[2,8], Sven Falke[1], Tian Geng[9], Tokushi Sato[2], Vasundara Srinivasan[7], Yoonhee Kim[2], Oleksandr M. Yefanov[1], Luca Gelisio[2], Tobias Beck[3,10], Andrew S. Doré[9,11], Adrian P. Mancuso[2,12,‡], Christian Betzel[7,10], Saša Bajt[1,10], Lars Redecke[13,14], Henry N. Chapman[1, 6, 10], Alke Meents[1], Dušan Turk[15,16], Winfried Hinrichs[17], Thomas J. Lane[1,10,11,#]

* these authors contributed equally to this work
\# correspondence: thomas.lane@desy.

1. Center for Free-Electron Laser Science CFEL,
Deutsches Elektronen-Synchrotron DESY,
Notkestr. 85, 22607 Hamburg, Germany

2. European XFEL GmbH,
Holzkoppel 4, 22869 Schenefeld, Germany

3. Universität Hamburg,
Department of Chemistry, Institute of Physical Chemistry
Grindelallee 117, 20146 Hamburg, Germany

4. Max Plank Institute for the Structure and Dynamics of Matter,
Luruper Chaussee 149, 22761 Hamburg, Germany

5. University of California at Irvine,
Department of Chemistry,
Irvine, CA 92697-2025, USA

6. Universität Hamburg,
Department of Physics,
Luruper Chaussee 149, 22761 Hamburg, Germany

7. Universität Hamburg
Department of Chemistry, Institute of Biochemistry and Molecular Biology,
Laboratory for Structural Biology of Infection and Inflammation,
Build. 22a, c/o DESY, Notkestr. 85, 22607 Hamburg, Germany

8. Gwangju Institute of Science and Technology,
123 Cheomdangwagi-ro, Buk-gu, Gwangju, 61005, Republic of Korea

9. Sosei Heptares,
Steinmetz Building, Granta Park, Great Abington, Cambridge CB21 6DG, United Kingdom

10. The Hamburg Centre for Ultrafast Imaging,
Luruper Chaussee 149, 22761 Hamburg, Germany

11. CHARM Therapeutics Ltd.
B900 Babraham Research Campus,
Cambridge CB22 3AT, United Kingdom

12. La Trobe University
Department of Chemistry and Physics, La Trobe Institute for Molecular Science,
Melbourne, Victoria 3086, Australia

13. Universität zu Lübeck, Institute of Biochemistry,
Ratzeburger Allee 160, 23562 Lübeck, Germany

14. Deutsches Elektronen-Synchrotron DESY,
Notkestr. 85, 22607 Hamburg, Germany

15. Jožef Stefan Institute,
Jamova cesta 39, 1000 Ljubljana European, Slovenija

16. Centre of excellence for Integrated Approaches in Chemistry and Biology of Proteins
Jamova 39, 1 000 Ljubljana, Slovenia

17. Universität Greifswald,
Institute of Biochemistry,
Felix-Hausdorff-Str. 4, 17489 Greifswald, Germany

‡ Present Address:
Diamond Light Source, Harwell Science and Innovation Campus,
Didcot OX11 0DE, United Kingdom

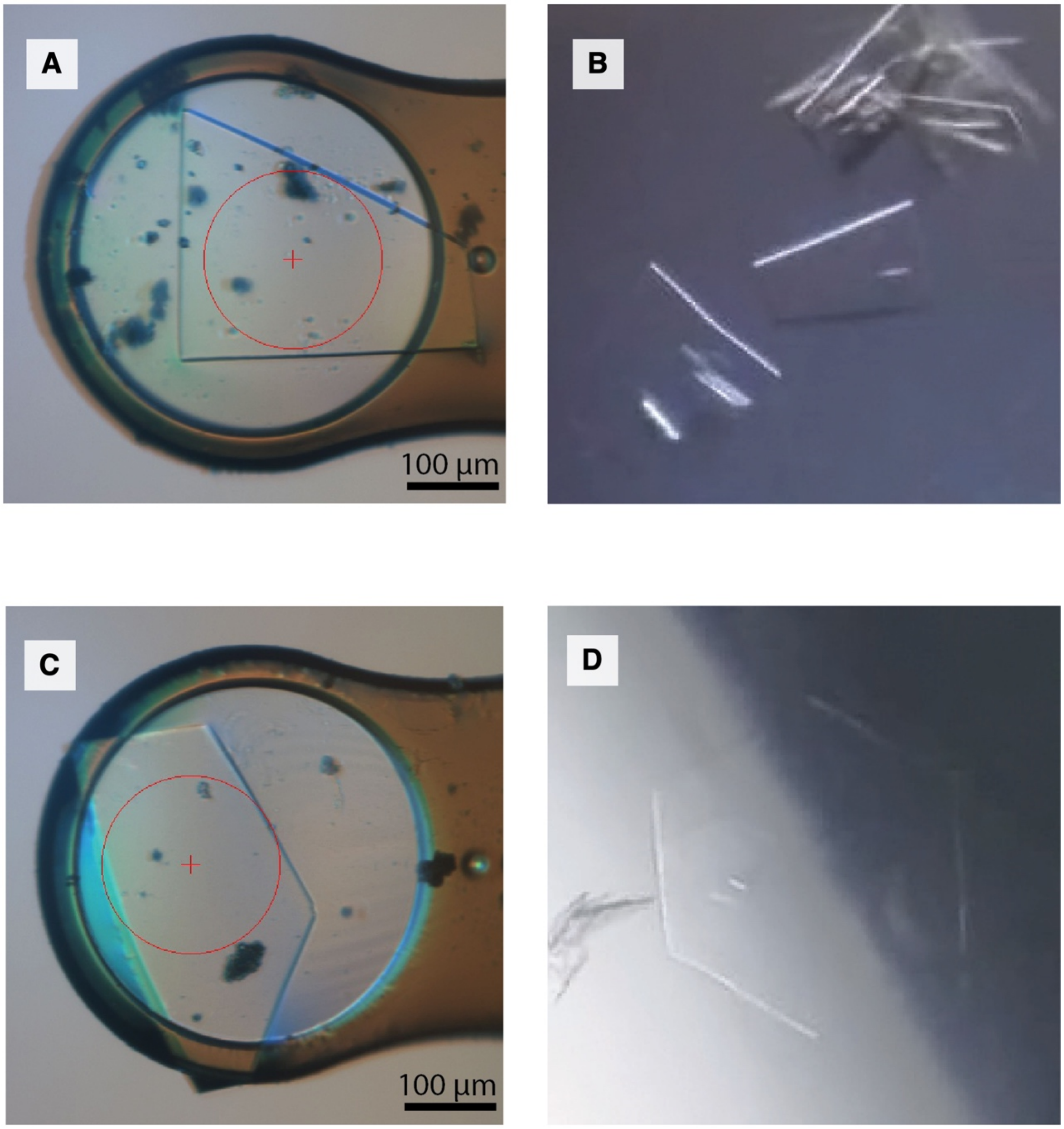

Figure S1. Crystal morphology of M^{pro} crystallized in two space groups, grown under the same conditions but with different seeds. (**A**, **B**) Reduced M^{pro} crystals in the monoclinic (C2) space group, as (**A**) a single fished crystal in a loop and (**B**) growing in a sitting-well drop. Crystals are approximately 20 µm thick. (**C**, **D**) Seeding with oxidized, orthorhombic seeds produced M^{pro} crystals in an orthorhombic ($P2_12_12_1$) space group, shown (**C**) in a loop and (**D**) in sitting drop. These crystals are approximately 10 µm thick, thinner than their monoclinic counterparts. The oxidized (*cf.* 7PZQ) and reduced (*cf.* 7Z2K) orthorhombic crystals are morphologically indistinguishable; shown here are crystals without TCEP, exposed to air. Study with synchrotron radiation of such crystals thus far yielded ambiguous density at the C117-C145 disulfide site, with occasional residual but uninterpretable density. Only with XFEL radiation on microcrystalline versions of these crystals have we obtained density unambiguously supporting a disulfide bond.

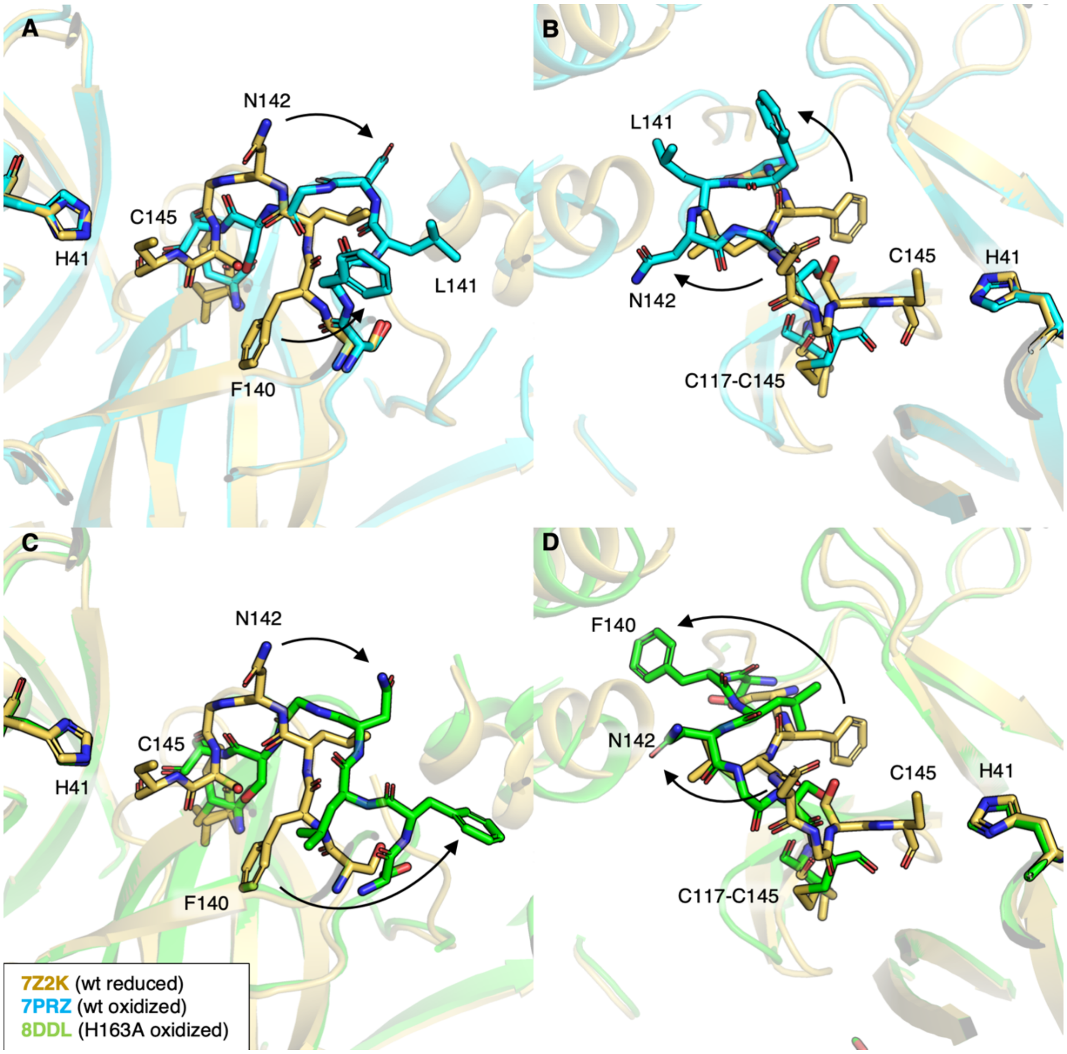

Figure S2. A comparison between C117-C145 in wild-type M^{pro} and the H163A variant, focusing on structure of the loop spanning S139 to S147. The proteolytic transition state is stabilized by an oxyanion hole formed by the backbone amides of G143, S144, and C145, as well as the hydroxyl side chain of S144. These residues form a loop that is restructured in both the wild-type enzyme exhibiting the C117-C145 disulfide (cyan, PDB ID: 7PRZ) as well as a previously reported structure of M^{pro} H163A with the same disulfide (green, PDB ID: 8DDL)[1]. Our reduced structure in the same space group is shown for comparison in all panels (yellow, PDB ID: 7Z2K). The loop containing F140 is displaced from the reduced position in both oxidized structures but is in distinct conformations in these two structures. (**A**, **B**) In the wild-type oxidized structure, the loop is partially ordered, and was modeled in two different conformations. Moderate loop rearrangement is necessary to accommodate the large displacement needed to bring C117 and C145 together. (**C**, **D**) In the H163A variant, however, a π-stacking interaction between H163 and F140 is missing. As a result, F140 and L141 undergo dramatic displacement, flipping outward from their reduced positions towards the dimeric protomer. In both oxidized cases, disruption of the N and C termini from their reduced positions is apparent, resulting in a less tightly packed dimer interface.

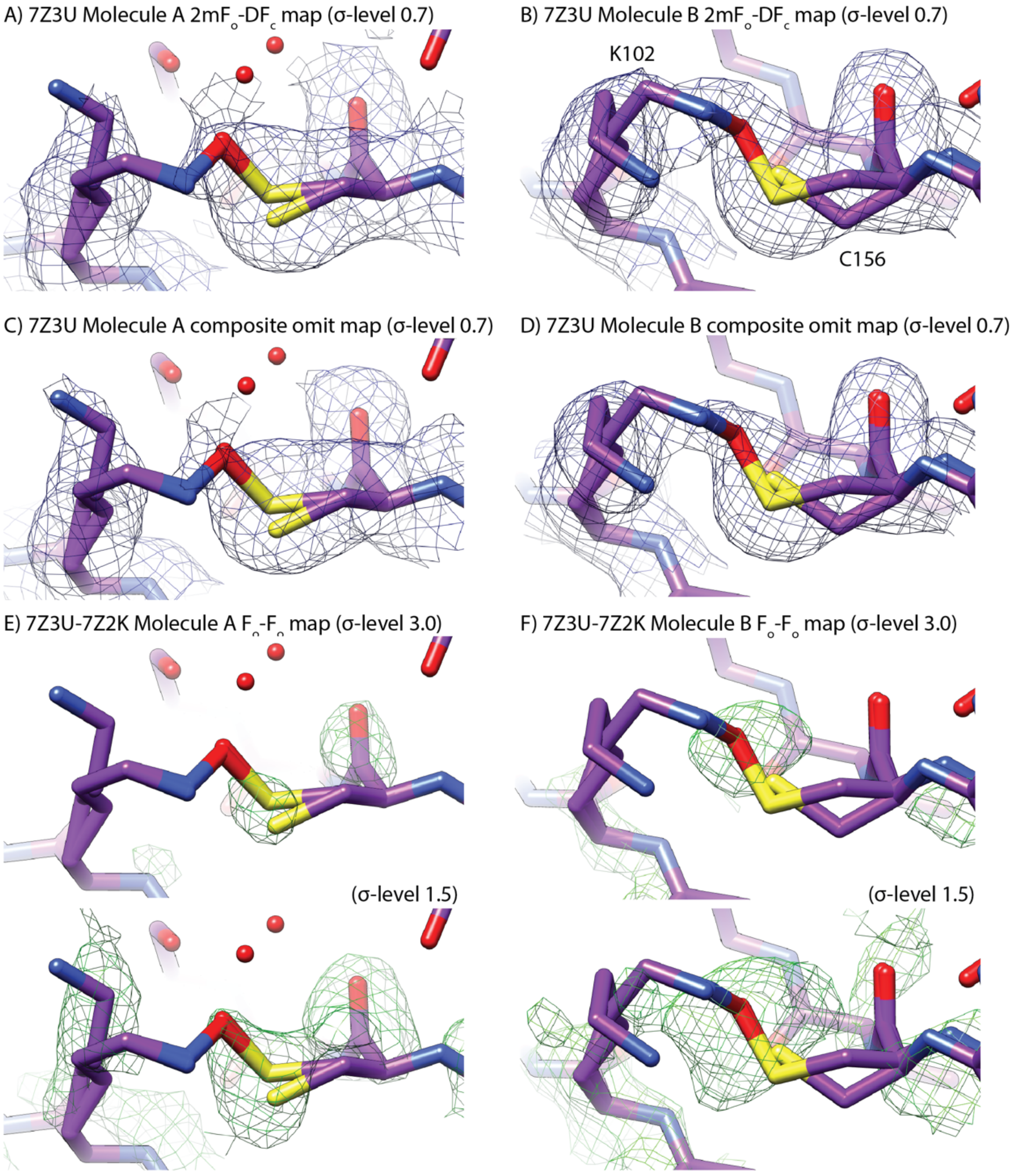

Figure S3. Crystallographic analysis of the K102/C156 NOS linkage. Three types of map were used to interrogate the presence or absence of density suggesting a NOS linkage between K102 and C156. We computed (**A**, **B**) traditional $2mF_o$-DF_c maps (0.7σ), (**C**, **D**) composite OMIT maps (0.7σ), and (**E**, **F**) isomorphous difference maps ($+3\sigma$, top and $+1.5\sigma$, bottom) for the amplitude difference between PDB ID 7Z3U (oxidized/calpeptin) and 7Z2K (reduced) with phases from 7Z2K. Both chain A (panels A, C, E) and chain B (panels B, D, F) are shown. While there is some density between K102 and C156 for chain A, it is not possible to confidently conclude a NOS bond exists. In contrast, for chain B, all three map types show evidence of a NOS linkage at partial occupancy.

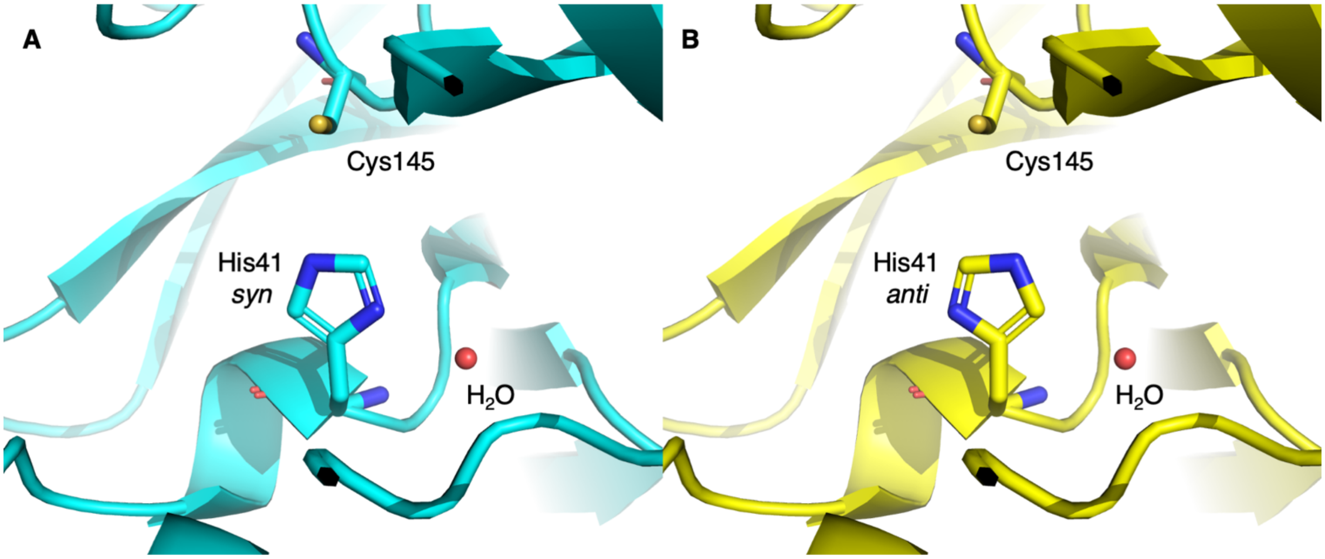

Figure S4. Definition used to describe conformer of His41. (**A**) In the *syn* conformation (cyan), histidine N_δ is proximal to a conserved and strongly bound water in the active site. (**B**) The *anti* conformer (yellow) is produced by a 180° flip of the sidechain around the C_β-C_γ bond. In both cases the catalytic water exhibits hydrogen bonds to His164(ND1), Asp187(OD2), and His41(N).

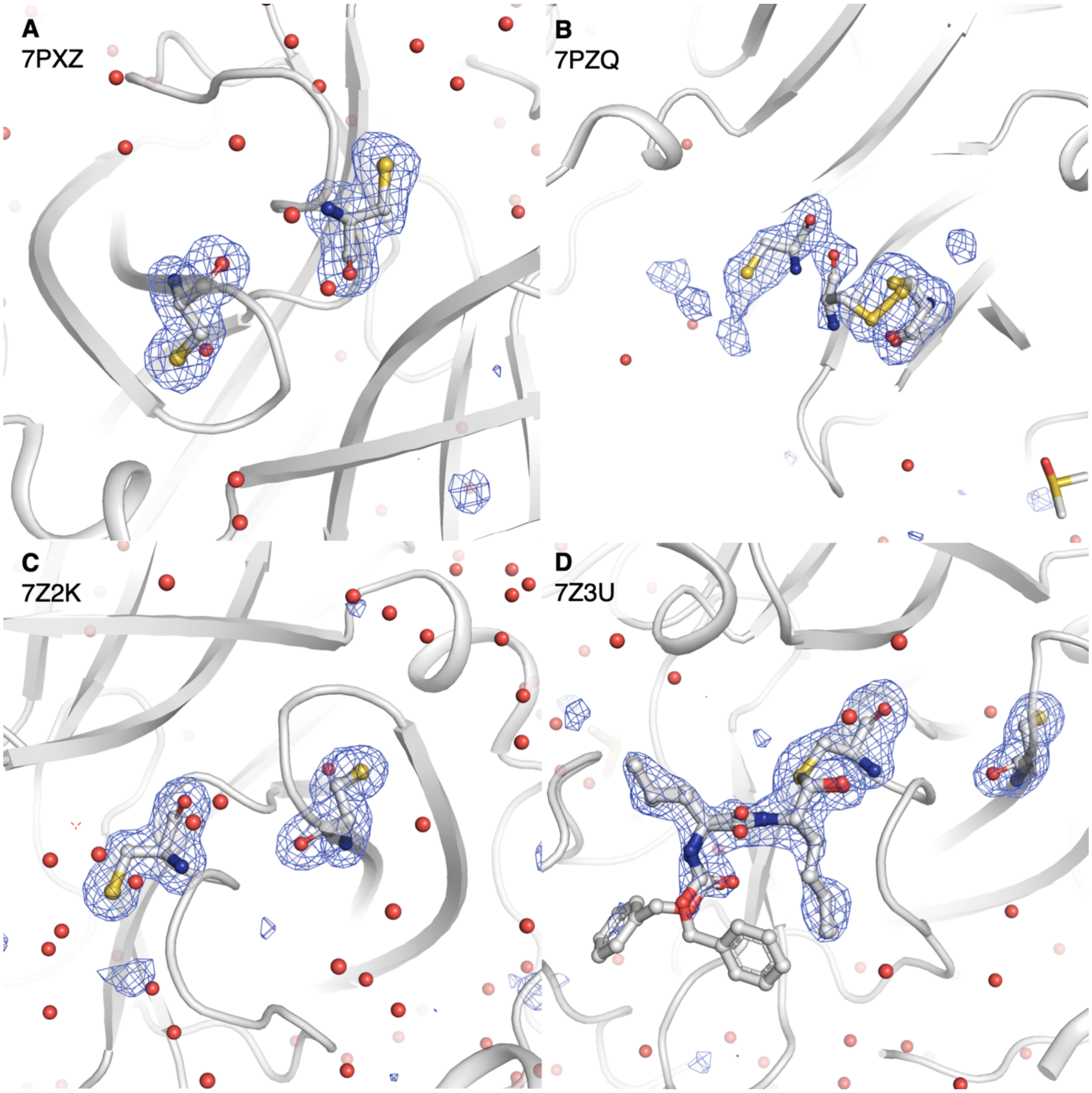

Figure S5. Polder omit maps of C145 and C117 for each structure presented. Shown are polder-style OMIT maps contoured at +4 σ, for C117, C145, and S-calpeptin omitted from the phase calculation. Note S-calpeptin is only present and relevant for PDB ID 7Z3U (panel D). Maps are not clipped. C117, C145 and S-calpeptin shown as sticks, all other residues shown as cartoon, waters shown as spheres.

Table S1. Data collection and refinement statistics

M^{pro}	Reduced Monoclinic+	Oxidized (S-S) Orthorhombic†	Reduced Orthorhombic	Oxidized (NOS/SONOS) Orthorhombic
**PDB ID**	7PXZ	7PZQ	7Z2K	7Z3U‡
**Data collection**				
Source	EuXFEL	EuXFEL	PETRA-III	PETRA-III
Temperature	297 K	297 K	100 K	100 K
Space group	C2	$P2_12_12_1$	$P2_12_12_1$	$P2_12_12_1$
Cell dimensions				
a, *b*, *c* (Å)	115.0 54.0 45.0	104.4 104.4 68.7	67.8 101.0 103.9	67.7 99.6 103.261
α, β, γ (°)	90 102.0 90	90 90 90	90 90 90	90 90 90
Solvent Content (%)	38.1	56.04	51.34	50.34
Resolution (Å)	31.62-1.75 (1.81-1.75)	24.61-2.25 (2.33-2.25)	67.76-1.65 (1.71-1.65)	49.22-1.72 (1.782-1.72)
Wilson B (Å^2)	24.9	28.6	22.7	23.7
R_{sym}[1], R_{split}[2]	0.071 (1.195)[2]	0.169 (3.117)[2]	0.047 (0.576)[1]	0.039 (0.828)[1]
$I / \sigma I$	11.44 (0.72)	7.41 (0.04)	23.40 (1.26)	12.54 (0.95)
$CC_{1/2}$	0.996 (0.207)	0.979 (0.29)	0.999 (0.598)	0.999 (0.524)
CC^*	0.999 (0.59)	0.995 (0.67)	0.999 (0.865)	0.999 (0.829)
Completeness (%)	99.52 (95.52)	99.88 (99.86)	99.34 (98.46)	99.70 (98.31)
Redundancy	946.0 (24.0)	355.6 (5.4)	6.9 (6.6)	7.5 (7.7)
**Refinement**				
Resolution (Å)	31.62-1.75 (1.813-1.75)	24.61-2.25 (2.33-2.25)	67.76-1.65 (1.71-1-65)	49.22-1.72 (1.782-1.72)
No. reflections	27225 (2602)	36330 (3592)	85170 (1993)	74542 (7273)
R_{work} / R_{free}	0.164 / 0.212	0.176 / 0.243	0.185 / 0.213	0.191 / 0.235
No. atoms	2724	5058	5590	5440
Protein	2490	4819	4945	4911
Ligand/ion	1	30	41	205
Water	233	227	624	435
B-factors				
Protein (Å^2)	35.5	38.1	29.1	38.1
Ligand/ion (Å^2)	21.4	58.1	42.7	52.6
Water (Å^2)	49.8	39.4	37.3	43.6
r.m.s. deviations				
Bond lengths (Å)	0.012	0.013	0.011	0.013
Bond angles (°)	1.26	1.22	1.14	1.28

+ Number of crystals merged (serial): 214 954

† Number of crystals merged (serial): 41 771

‡ As reported in 2

		7PXZ		7PZQ*		7Z2K		7Z3U*	
**chain**		***syn***	*anti*	*syn*	***anti***	*syn*	***anti***	*syn*	***anti***
**A**	H41 CG B factor (Å^2)	24.4	23.3	18.6	19.3	18.2	19.2	29.7	30.4
	H41 ND1 B factor (Å^2)	29.5	36.8	25.9	23.4	24.6	23.3	33.7	39.4
	H41 CD2 B factor (Å^2)	32.6	25.0	19.9	22.3	19.0	20.9	34.7	30.6
	H41 CE1 B factor (Å^2)	24.8	32.2	20.6	27.6	21.2	24.9	36.1	36.6
	H41 NE2 B factor (Å^2)	34.7	27.9	31.1	23.5	29.0	24.8	38.4	39.0
	Total sidechain B (Å^2)	146.0	145.2	116.1	116.1	112.0	113.1	172.6	176.0
	ND-SG distance (Å)	5.2	5.2	13.0	12.4	5.2	5.1	5.0	5.2
	NE-SG distance (Å)	3.8	3.8	11.3	11.7	3.7	3.8	3.7	3.6
				7PZQ*		7Z2K		7Z3U*	
				syn	*anti*	*syn*	***anti***	*syn*	***anti***
**B**	H41 CG B factor (Å^2)			32.4	33.2	19.5	19.0	31.9	32.4
	H41 ND1 B factor (Å^2)			38.3	32	27.3	26.1	30.0	26.4
	H41 CD2 B factor (Å^2)			29.2	34.3	22.1	22.5	22.5	26.0
	H41 CE1 B factor (Å^2)			35.1	34.7	17.9	26.8	22.2	28.7
	H41 NE2 B factor (Å^2)			38.6	37.6	29.6	21.6	31.1	25.7
	Total sidechain B (Å^2)			173.6	171.8	116.4	116.0	137.7	139.2
	B ND-SG distance (Å)			12.9	12.5	5.2	5.1	5.1	5.2
	B NE-SG distance (Å)			11.3	11.7	3.7	3.8	3.8	3.7

syn = His N_δ proximal to conserved H_2O, *anti* = 180° rotamer flip, see Fig. S4.
*structure in which C145 is involved in an inactivating covalent bond

Table S2. Assessment of rotameric state of H41 by refined imidazole sidechain. Shown are the B factors and N-to-S_γ distances for the catalytic dyad, H41 and C145 refined in both the *syn* and *anti* conformations (Fig. S4). Total B factors for these two conformers are very close, suggesting our data do not significantly favor one conformer over the other, and that both may be populated in superposition. The distances between H41 N_ε and C145 S_γ are not substantially different in one conformer *vs.* the other. Thus, both conformers are within the necessary hydrogen bonding distance between imidazolium 41 and thiolate 145 to produce the initial state of the catalytic cycle. In the absence of other information, we chose the *syn* or *anti* conformation based on the individual B factors of corresponding imidazole C/N-atom pairs, *e.g.* the B factor of NE2 should not be much higher than CE1. The final modeled rotameric state is indicated with bold font. Note that in 7PZQ and 7Z3U that Cys145 is in a disulfide and thiohemiacetal form, respectively, and therefore not capable of catalysis.

Supplemental References

1. Tran, N. *et al.* The H163A mutation unravels an oxidized conformation of the SARS-CoV-2 main protease. *Nat Commun* **14**, (2023).
2. Reinke, P. Y. A. *et al.* Calpeptin is a potent cathepsin inhibitor and drug candidate for SARS-CoV-2 infections. *Commun Biol* **6**, (2023).
