# Supplementary material for: SARS-CoV-2 Mpro responds to oxidation by forming disulfide and NOS/SONOS bonds
Source: Nat Commun. 2024 May 7;15:3827. doi: 10.1038/s41467-024-48109-3 (PMC11076503; doi:10.1038/s41467-024-48109-3)
Supplement: Supplementary file 3 — Reporting Summary [file 41467_2024_48109_MOESM3_ESM.pdf]

Corresponding author(s): LANE, Thomas

Last updated by author(s): 25 March 2024

Reporting Summary

Nature Portfolio wishes to improve the reproducibility of the work that we publish. This form provides structure for consistency and transparency in reporting. For further information on Nature Portfolio policies, see our Editorial Policies and the Editorial Policy Checklist.

Statistics

For all statistical analyses, confirm that the following items are present in the figure legend, table legend, main text, or Methods section.

n/a	Confirmed	
☐	☒	The exact sample size (*n*) for each experimental group/condition, given as a discrete number and unit of measurement
☒	☐	A statement on whether measurements were taken from distinct samples or whether the same sample was measured repeatedly
☒	☐	The statistical test(s) used AND whether they are one- or two-sided<br>*Only common tests should be described solely by name; describe more complex techniques in the Methods section.*
☒	☐	A description of all covariates tested
☒	☐	A description of any assumptions or corrections, such as tests of normality and adjustment for multiple comparisons
☒	☐	A full description of the statistical parameters including central tendency (e.g. means) or other basic estimates (e.g. regression coefficient) AND variation (e.g. standard deviation) or associated estimates of uncertainty (e.g. confidence intervals)
☒	☐	For null hypothesis testing, the test statistic (e.g. *F*, *t*, *r*) with confidence intervals, effect sizes, degrees of freedom and *P* value noted<br>*Give P values as exact values whenever suitable.*
☒	☐	For Bayesian analysis, information on the choice of priors and Markov chain Monte Carlo settings
☒	☐	For hierarchical and complex designs, identification of the appropriate level for tests and full reporting of outcomes
☒	☐	Estimates of effect sizes (e.g. Cohen's *d*, Pearson's *r*), indicating how they were calculated

Our web collection on statistics for biologists contains articles on many of the points above.

Software and code

Policy information about availability of computer code

Data collection	During data collection at the European XFEL, we employed the online monitoring codes Karabo (v2) and OnDA (v22), provided by the facility.
Data analysis	Crystallographic analysis was performed with cheetah (v2019.1), CrystFEL (v0.9.1), XDS (v 10.01.2022), phenix (v1.20.1), PISA (v1.48) and Coot (v0.9) Analytical SEC data was analyzed using custom scripts, available along with raw data via Zenodo (10.5281/zenodo.10616060).

For manuscripts utilizing custom algorithms or software that are central to the research but not yet described in published literature, software must be made available to editors and reviewers. We strongly encourage code deposition in a community repository (e.g. GitHub). See the Nature Portfolio guidelines for submitting code & software for further information.

Data

Policy information about availability of data

All manuscripts must include a data availability statement. This statement should provide the following information, where applicable:

- Accession codes, unique identifiers, or web links for publicly available datasets
- A description of any restrictions on data availability
- For clinical datasets or third party data, please ensure that the statement adheres to our policy

Structural models, structure factor data, and associated metadata are available from the Protein DataBank under PDB IDs 7PXZ, 7PZQ, 7Z2K & 7Z3U. Raw diffraction data available via EuXFEL (https://doi.org/10.22003/XFEL.EU-DATA-002696-00). Analytical SEC data and processing code are available via Zenodo (10.5281/zenodo.10616060).

Research involving human participants, their data, or biological material

Policy information about studies with human participants or human data. See also policy information about sex, gender (identity/presentation), and sexual orientation and race, ethnicity and racism.

Reporting on sex and gender	N/A
Reporting on race, ethnicity, or other socially relevant groupings	N/A
Population characteristics	N/A
Recruitment	N/A
Ethics oversight	N/A

Note that full information on the approval of the study protocol must also be provided in the manuscript.

Field-specific reporting

Please select the one below that is the best fit for your research. If you are not sure, read the appropriate sections before making your selection.

☒ Life sciences ☐ Behavioural & social sciences ☐ Ecological, evolutionary & environmental sciences

For a reference copy of the document with all sections, see nature.com/documents/nr-reporting-summary-flat.pdf

Life sciences study design

All studies must disclose on these points even when the disclosure is negative.

Sample size	Serial crystallographic data was collected according to current best practice (214 954 and 41 771 indexed images for the two relevant datasets), see Gorel, Schlichting and Barends IUCrJ 8, 532-543 (2021). Crystals were reported for rotational data collection for which merging cross-correlations exceed 0.5 for the resolution range reported. SEC experiments are reported as a concentration series. To span the relevant KDs by an order of magnitude by no less than an order of magnitude, we employed 8 serial dilution by factors of 2 (2^8 =256).
Data exclusions	No data were manually excluded.
Replication	Analytical SEC experiments were replicated no fewer than 3 times. While the results are qualitatively consistent, the data quality of earlier experiments was not sufficient to include in publication. Serial crystallographic results were confirmed by quality by dividing the datasets into two and performing cross-correlation (CC* > 0.5 for all reported intensities) ensuring statistical reproducibility. For rotation data collection, no fewer than 3 crystals were collected per condition, but only the highest quality dataset showing unusual features (specifically NOS/SONOS bonds) were analyzed in detail as described in the paper.
Randomization	Experiments involved the biophysical characterization of recombinant protein (Mpro) for which randomization is not applicable.
Blinding	Experiments involved the biophysical characterization of recombinant protein (Mpro) for which blinding is not applicable.

Reporting for specific materials, systems and methods

We require information from authors about some types of materials, experimental systems and methods used in many studies. Here, indicate whether each material, system or method listed is relevant to your study. If you are not sure if a list item applies to your research, read the appropriate section before selecting a response.

Materials & experimental systems

n/a	Involved in the study	
☒	☐	Antibodies
☒	☐	Eukaryotic cell lines
☒	☐	Palaeontology and archaeology
☒	☐	Animals and other organisms
☒	☐	Clinical data
☒	☐	Dual use research of concern
☒	☐	Plants

Methods

n/a	Involved in the study	
☒	☐	ChIP-seq
☒	☐	Flow cytometry
☒	☐	MRI-based neuroimaging

Plants

Seed stocks	*Report on the source of all seed stocks or other plant material used. If applicable, state the seed stock centre and catalogue number. If plant specimens were collected from the field, describe the collection location, date and sampling procedures.*
Novel plant genotypes	*Describe the methods by which all novel plant genotypes were produced. This includes those generated by transgenic approaches, gene editing, chemical/radiation-based mutagenesis and hybridization. For transgenic lines, describe the transformation method, the number of independent lines analyzed and the generation upon which experiments were performed. For gene-edited lines, describe the editor used, the endogenous sequence targeted for editing, the targeting guide RNA sequence (if applicable) and how the editor was applied.*
Authentication	*Describe any authentication procedures for each seed stock used or novel genotype generated. Describe any experiments used to assess the effect of a mutation and, where applicable, how potential secondary effects (e.g. second site T-DNA insertions, mosiacism, off-target gene editing) were examined.*
